# C3a and C5b-9 Differentially Predict COVID-19 Progression and Outcome

**DOI:** 10.3390/life12091335

**Published:** 2022-08-28

**Authors:** Maria G. Detsika, Elpida Diamanti, Kleio Ampelakiotou, Edison Jahaj, Stamatis Tsipilis, Nikolaos Athanasiou, Ioanna Dimopoulou, Stylianos E. Orfanos, Alexandra Tsirogianni, Anastasia Kotanidou

**Affiliations:** 1 1st Department of Critical Care Medicine & Pulmonary Services, GP Livanos and M Simou Laboratories, Evangelismos Hospital, National and Kapodistrian University of Athens, 10675 Athens, Greece; 2 Department of Immunology and Histocompatibility, Evangelismos General Hospital, 10676 Athens, Greece

**Keywords:** COVID-19, complement, biomarkers, mortality

## Abstract

SARS-CoV-2 infection may result in severe pneumonia leading to mechanical ventilation and intensive care (ICU) treatment. Complement activation was verified in COVID-19 and implicated as a contributor to COVID-19 pathogenesis. This study assessed the predictive potential of complement factors C3a and C5b-9 for COVID-19 progression and outcome. We grouped 80 COVID-19 patients into severe COVID-19 patients (n = 38) and critically ill (n = 42) and subdivided into non-intubated (n = 48) and intubated (n = 32), survivors (n = 57) and non-survivors (n = 23). Results: A significant increase for C3a and C5b-9 levels was observed between: severely and critically ill patients (*p* < 0.001 and *p* < 0.0001), non-intubated vs intubated (*p* < 0.001 and *p* < 0.05), survivors vs non-survivors (*p* < 0.001 and *p* < 0.01). ROC analysis for the need for ICU treatment revealed a higher AUC for C5b-9 (0.764, *p* < 0.001) compared to C3a (AUC = 0.739, *p* < 0.01). A higher AUC was observed for C3a for the need for intubation (AUC = 0.722, *p* < 0.001) or mortality (AUC = 0.740, *p* < 0.0001) compared to C5b-9 (need for intubation AUC = 0.656, *p* < 0.05 and mortality AUC = 0.631, *p* = NS). Combining the two markers revealed a powerful prediction tool for ICU admission (AUC = 0.773, *p* < 0.0001), intubation (AUC = 0.756, *p* < 0.0001) and mortality (AUC = 0.753, *p* < 0.001). C3a and C5b-9 may be considered as prognostic tools separately or in combination for the progression and outcome of COVID-19.

## 1. Introduction

Three years since the beginning of the coronavirus disease 2019 (COVID-19) pandemic, COVID-19 remains a threat to health systems, with waves of infection emerging unpredictably. Therefore, swift and accurate patient assessment is essential to sustain successful management and treatment of patients admitted to hospitals or intensive care units (ICU).

Although the exact mechanisms driving COVID-19 pathogenicity remain to be identified, it is widely known that SARS-CoV-2 infection in some cases, results in a dysregulated immune response leading to the release of pro-inflammatory cytokines (cytokine storm) accompanied by increased thrombosis and coagulation [1,2,3].

The complement system is an immune mechanism in the first line of defense against pathogens, including viruses. Activation of the complement system occurs mainly through three pathways: (i) the classical pathway, in which antibodies bound to antigen, recruit the C1 complex, which via activation of circulating C4 and C2 increases the rate of C3 cleavage at the antibody-coated surface, (ii) the mannose-binding lectin (MBL) pathway, in which mannose residues on microbial surfaces are recognized by MBL proteins, also leading to recruitment and activation of C4 and C2 and (iii) the alternative pathway, which is permanently active at a low level due to spontaneous transformation of C3 to C3(H_2_O). Hydrolyzed C3 subsequently undergoes structural changes exposing a binding site for Factor B (FB). Upon binding to FB, cleavage by factor D (FD) results in generating the alternative pathway C3 convertase complex C3(H_2_O)Bb. The three pathways converge at the C3 step, at which C3 cleavage by the C3 convertases generates active C3b. The binding of C3b to the C3 convertases results in the formation of the C5 convertase, which promotes cleavage of circulating C5 into C5a and C5b. C5b generation activates the terminal pathway, in which C6–C9 are recruited to form the membrane attack complex (C5b-9), a pore-like structure causing lysis of targeted cells [4,5,6]. Moreover, C3a and C5a recruit and activate cells of the immune system.

Activation of the complement cascade has been verified in COVID-19 at circulation [7] and tissue levels [8]. Reports have linked the activation of all three complement cascade pathways throughout infection and COVID-19 progression [9,10]. The documented activation of all three complement cascade pathways during COVID-19, especially in patients with heavily thrombotic profiles [8], suggests a possible role of complement activation in disease progression and outcome. This study assessed C3a and C5b-9 levels to predict COVID-19 patient progression and outcome.

## 2. Materials and Methods

This study was reviewed by the Institutional Ethics Committee and approved the need for approval (Board name: “Evangelismos” Hospital Research Ethics Committee, approval number: 360, approval date: 17–9–2020, Study title: COVID-19 and Immunological profile). All procedures carried out followed the Helsinki Declaration. Informed written consent was obtained from all patients or the patient's next-of-kin. A total of 80 patients were included. All samples were obtained on the admission of patients. Patients were grouped into those with severe (n = 38) and critical (n = 42) disease. Patients were subsequently subdivided into non-intubated (n = 48) and intubated (n = 32) as well as survivors (n = 57) and non-survivors (n = 23).

### 2.1. C3a and C5b-9 Measurement by Standard Enzyme-Linked Immunosorbent Assay (ELISA) Methodology

As previously described, blood samples were collected and processed to assess C3 and C5b-9 levels [11]. All blood samples were collected in EDTA-coated tubes and stored at room temperature until further processing. All samples were processed within one hour following collection. For plasma isolation, blood samples were centrifuged for 10 min at 1000 *g* at 4 °C. Plasma samples were stored immediately at −80 °C until further use. Plasma samples were thawed at room temperature and stored on ice prior to loading. Standard ELISA methodology measured levels of C3a and C5b-9 in serum samples. C3a and C5b-9 ELISA kits were obtained from Quidel (San Diego, CA, USA).

### 2.2. Statistical Analysis

As appropriate, results are reported as absolute numbers, medians, or means and standard deviations. Statistical analysis was performed using the GraphPad Prism 8.0 software for Windows. Data were tested for normality using the Shapiro-Wilks test. Unpaired *t*-test or Mann–Whitney was used in the case of data displaying normality or not, respectively. Spearman correlation was used for the correlation of data. Receiver operating characteristic (ROC) analysis was performed using ICU admission, intubation, or survival as the classification variable, while C3a and/or C5b-9 levels on admission were used as prognostic variables. The optimal cut-off value for predicting the different factors was calculated as the point with the greatest combined sensitivity and specificity. A p-value *p* < 0.05 was considered statistically significant.

## 3. Results

### 3.1. Increase of C3a and C5b-9 Levels in COVID-19 Patients

Patient demographic data are shown in Table 1. Patient groups showed differences in line with the characteristics of the disease but had no differences in terms of comorbidities such as diabetes, cardiovascular disease and chronic obstructive pulmonary disease (COPD).

**Figure 1:**
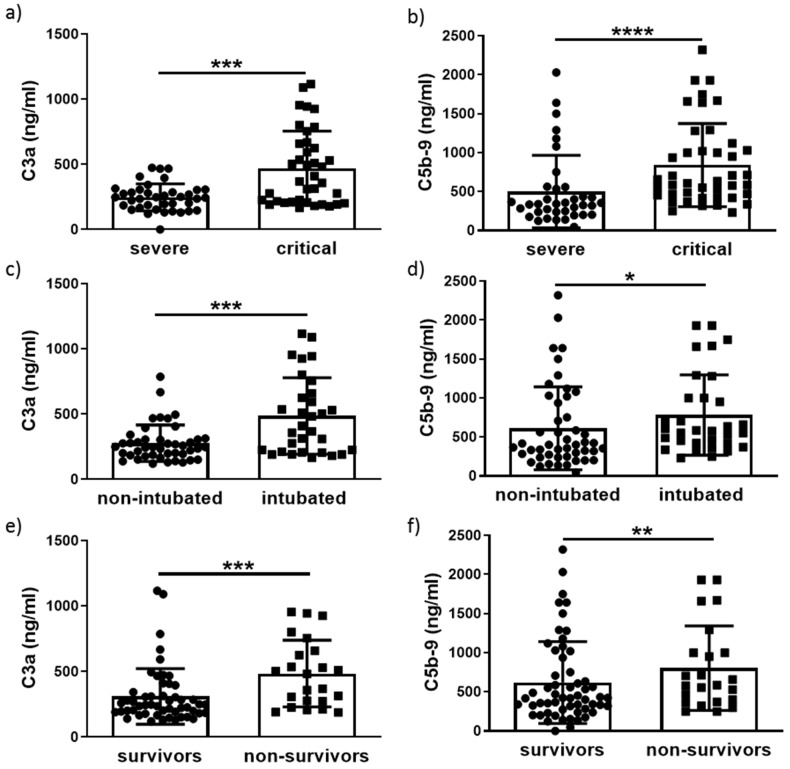
Increased levels of C3a and C5b-9 in COVID-19 patients. C3a and C5b-9 levels measured on admission were higher in critically ill compared to patients with severe COVID-19 (**a**,**b**), in intubated versus non-intubated (**c**,**d**), and in non-survivors compared to survivors (**e**,**f**). Data are expressed as means ± SD. Statistical analysis was performed using the Mann–Whitney U test. * *p* < 0.05, ** *p* < 0.01, *** *p* < 0.001, **** *p* < 0.0001.

**Figure 2:**
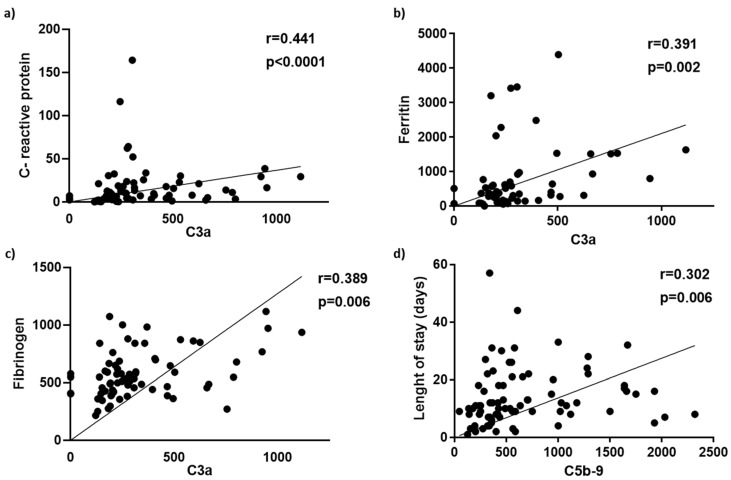
C3a and C5b-9 levels correlations in COVID-19 disease. C3a levels correlated positively with (**a**) C-reactive protein, (**b**) ferritin and (**c**) fibrinogen levels. (**d**) A positive correlation between C5b-9 levels with patient hospital length of stay (ward/ICU).

A statistically significant increase of C3a (*p* < 0.001) and C5b-9 (*p* < 0.0001) levels was observed in critically ill patients compared to those with severe disease (Figure 1a,b). Further grouping into intubated and non-intubated revealed a significant increase for both C3a (*p* < 0.001) and C5b-9 (*p* < 0.05) in intubated versus non-intubated patients (Figure 1c,d). C3a and C5b-9 levels remained significantly elevated in non-survivors (*p* < 0.001) compared to survivors (*p* < 0.01) (Figure 1e,f). Spearman correlation revealed a positive correlation of C3a with CRP (r = 0.442, *p* < 0.0001), ferritin levels (r = 0.391, *p* = 0.002) and fibrinogen (r = 0.389, *p* = 0.006) and a weak but positive correlation of C5b-9 with patient length of stay (LOS) in either ward or ICU (r = 0.302, *p* = 0.007) (Figure 2).

### 3.2. Differential Prediction of COVID-19 Progression and Outcome by C3a and C5b-9 Levels

**Figure 3:**
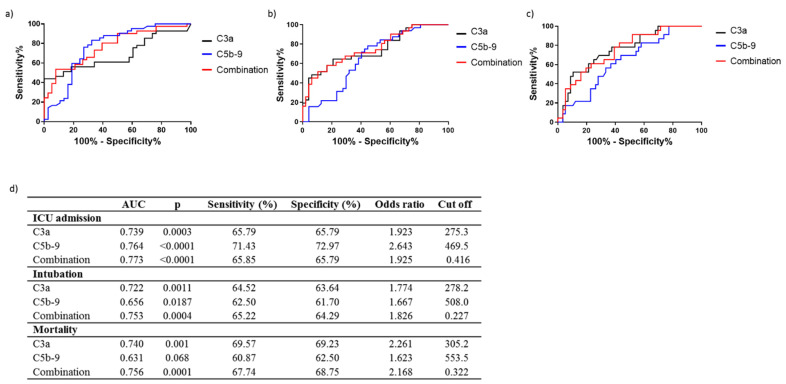
Increased levels of C3a and C5b-9 in COVID-19 patients differentially predict ICU admission, intubation and mortality. Receiver operating characteristic curves for prediction of need for intensive care treatment (**a**) intubation (**b**) and survival (**c**). (**d**) Corresponding area under the curve (AUC), *p* values, and optimal cut-off points with combined greatest sensitivity (%) and specificity (%) and odds ratio values are shown.

The prognostic accuracy of C3a and C5b-9 for prediction of ICU admission, need for intubation, and survival was next determined by ROC analysis (Figure 3). The area under the curve (AUC) for prediction of ICU admission was 0.739 (95% CI = 0.627–0.852, *p* = 0.0003) and 0.764 (95% CI = 0.654–0.875, *p* < 0.0001) for C3a and C5b-9, respectively (Figure 3c,d). Similarly, AUC for the need for intubation for C3a was 0.722, (95% CI = 0.601–0.844, *p* = 0.0011) compared to 0.656, (95% CI = 0.537–0.776, *p* = 0.018) for C5b-9 (Figure 3c,d). C3a AUC for mortality prediction was 0.740, (95% CI = 0.620–0.860, *p* = 0.0010) compared to C5b-9 which proved a weak candidate for mortality prediction with an AUC of 0.631 and a non-significant p-value (95% CI = 0.530–0.759, *p* = 0.068) (Figure 3c,d). Combination of C3a and C5b-9 resulted in an AUC of 0.773, 95% CI = 0.670–0.874, *p* < 0.0001 for ICU admission, 0.753, 95% CI = 0.640–0.865, *p* = 0.0004 for intubation and 0.756, 95% CI = 0.646–0.865, *p* = 0.0001 for mortality (Figure 3a–d).

### 3.3. C3a and C5b-9 Performance as COVID-19 Prognosis Tools

**Figure 4:**
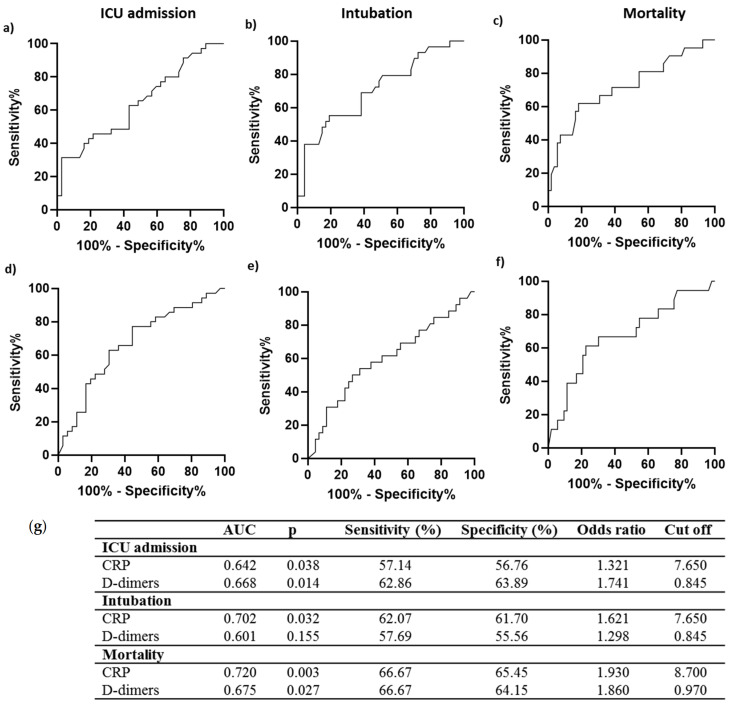
Prediction of ICU admission, intubation and mortality by C-reactive protein and D-dimers. Receiver operating characteristic (ROC) curves of C-reactive protein for prediction of need for intensive care (**a**) intubation (**b**) and mortality (**c**) and D-dimer levels (**d**–**f**). The corresponding areas under the curve (AUC), *p*, sensitivity and specificity, odd ratio, and cut-off values are represented in (**g**).

The prognostic accuracy of C3a and C5b-9 was further compared to that of C-reactive protein (CRP) and D-dimers, considered standard biomarkers for hyper-inflammation and coagulation, respectively, during COVID-19 [12]. The AUC values for prediction of ICU admission, intubation and mortality by C3a alone were higher compared to both CRP (ICU admission AUC = 0.642, 95% CI = 0.514–0.769, *p* = 0.038, Intubation AUC = 0.702, 95% CI = 0.579–0.825, *p* = 0.003, Mortality AUC = 0.720, 95% CI = 0.582–0.858, *p* = 0.003) and D-dimers (ICU admission AUC = 0.668, 95% CI = 0.541–0.795, *p* = 0.014, Intubation AUC = 0.601, 95% CI = 0.460–0.742, *p* = NS, Mortality AUC = 0.675, 95% CI = 0.524–0.826, *p* = 0.002) (Figure 3 and Figure 4). A comparison of the performance of C5b-9 showed a higher AUC for the prediction of ICU admission than that of both CRP and D-dimers. In comparison, AUC for intubation prediction was lower than that for CRP but higher than D-dimers AUC (Figure 3 and Figure 4). Mortality prediction by C5b-9 proved poor in comparison to both CRP and D-dimers (Figure 3 and Figure 4). Finally, a combination of C3a and C5b-9 showed higher AUC values than CRP and D-dimers for all parameters tested.

## 4. Discussion

The present study demonstrates the differential ability of complement factors C3a and C5b-9 for predicting disease progression, such as patient admission in ICU as well as a patient need for intubation, and finally, prediction of the outcome when defined as mortality (Figure 3). Therefore, C3a and C5b-9 may be considered markers for differential prediction of ICU admission, intubation and survival, while their combination results in a powerful prognostic tool. C3a showed a similarly high AUC for predicting all parameters examined in our analysis, while C5b-9 could accurately predict ICU admission and, to a lesser extent, intubation but not mortality. Furthermore, the combination of the two markers resulted in higher AUC values for ICU admission, intubation and mortality, indicating its high predictive potential as a prognostic tool. Increased levels of C3a and C5b-9 in critically ill patients with severe disease have been reported with no significant difference between survivors and non-survivors [13]. C3a has been reported previously as a strong marker for mortality prediction, but its ability to predict ICU admission and intubation was not assessed [14], while in another study that did not include critically ill patients, complement over-activation failed to predict disease progression [15]. Our observations further support the important role of complement activation in COVID-19 pathogenesis. The differential ability of C3a and C5b-9 to predict disease progression and mortality observed in our study indicates that this may be a result of the different roles of each factor in COVID-19 progression and possibly affected by both their position in the complement cascade as well as their individual effect on immune responses. The superiority of C3a for the prediction of mortality, observed in our study, in comparison to C5b-9, which failed to predict mortality, is in line with a previous study [14]. This indicates that the upstream position of C3a in the cascade possibly allows for its increased predictive potential both for mortality as well as for COVID-19 progression overall. C5a has also been reported to possess a potential for predicting COVID-19 [16,17,18], but the performance of the specific factor was not assessed in the present study.

C3a and C5b-9 levels are significantly elevated in critically ill patients at the point of ICU admission (Figure 1), suggesting that continuous complement activation occurs during SARS-CoV-2 infection. This is in line with previous studies which investigated levels of various complement factors in COVID-19 patients [13,14,15]. Although elevation of C3a levels has been reported even in mild COVID-19 patients compared to healthy controls, this increase was not statistically significant [14,16]. The same has been observed for C5b-9 [14]. C3a and C5b-9 significant changes, compared to healthy controls, were shown only for hospitalized patients in need of oxygenation [14]. However, the continuous activation of complements during the course of the disease supports the potential of complement factors as prognostic markers of the disease. We focused on C3a and C5b-9 due to the difference in their position in the cascade, C3a as an upstream candidate and C5b-9 as the terminal component. We identified a differential potential in their ability to predict disease progression and outcome. Furthermore, the increased complement activation observed in COVID-19 patients has an implied role of complement as a contributor to the pro-thrombotic profile of these patients. In this regard, studies have identified increased deposition of complement factors in tissue samples of COVID-19 patients with thrombotic lesions [8] both in the ICU and in the hospital. The continuous activation of the complement cascade allows for its use as a source of prognostic tools for COVID-19 disease progression. To this end, the present study revealed the advanced ability of C3a and C5b-9 to predict both COVID-19 progression and outcome.

Our study further included a comparison of C3a and C5b-9 with other standard markers for COVID-19, such as CRP and D-dimers, which have previously been utilized for treatment strategy initiation [19] during COVID-19 (Figure 4). Comparative analysis revealed the superiority of C3a for prediction of COVID-19 progression and mortality over both CRP and D-dimers, while C5b-9 was superior only for prediction of ICU admission over both CRP and D-dimers and for prediction of intubation when compared to D-dimers. Despite the increased prognostic potential of C3a and its combination with C5b-9 in COVID-19 progression, specific complement factors are not routinely measured in clinical practice; therefore, their use has not been implemented in our clinical setting. However, their strong association with disease progression and mortality is of particular importance as it supports the involvement of complement activation in COVID-19 pathogenesis and highlights the possible value of the complement cascade as a source of future therapeutic strategies for COVID-19.

Efforts to treat COVID-19 with complement targeted treatment strategies have already been explored with various complement inhibitors at both the C3 [20] and C5 [21], refs. [22,23] levels tested as well as C1 inhibitors [24] and MASP-2 [25]. Our results may provide useful data for similar studies to guide successful treatment by complement inhibitors in similar studies and support the need for further studies exploring therapeutic strategies against COVID-19 and mechanisms by which complement activation influences COVID-19.

## 5. Conclusions

C3a and C5b-9 may be used for differential prediction of COVID-19 progression and mortality. The strong performance of C3a and C5b-9 as predictors of COVID-19 progression and outcome support the important role of complement activation in COVID-19. Further studies to determine the role of complement in COVID-19 remain essential.

## Figures and Tables

**Table 1:** Patient clinical and demographic data.

	Severe COVID-19	Critical COVID-19	*p* Value
Age (years)	58.46 ± 15.80	65.65 ± 13.14	0.022
Male	21 (55.26%)	29 (76.31%)	0.154
Diabetes	3 (7.89%)	7 (18.42%)	0.398
Cardiovascular disease	2 (5.26%)	7 (18.42%)	0.206
Chronic obstructive pulmonary disease	1 (2.63%)	2 (5.26%)	0.840
Symptoms			
Temperature (>37.3 °C)	18 (47.36%)	22 (57.89%)	0.655
paO2/FIO2	320.20 ± 60.09	119.13 ± 67.71	<0.0001
Days of illness before admission	6.27 ± 2.60	6.41 ± 3.37	0.819
Laboratory baseline			
White blood cells	6887.00 ± 3749	13136 ± 1106	<0.0001
Neutrophils	69.44 ± 14.88	80.72 ± 15.67	<0.0001
Lymphocytes	25.80 ± 16.86	12.28 ± 12.89	<0.0001
Platelets	221216 ± 97386	236912 ± 125951	0.523
C-reactive protein	8.314 ± 6.993	13.76 ± 10.95	0.037
Troponin	29.14 ± 93.72	481.20 ± 1404	<0.0001
Urea	31.16 ± 18.40	59.00 ± 47.47	<0.0001
Creatinine	0.843 ± 0.196	1.414 ± 1.835	0.122
Aspartate aminotransferase	40.62 ± 33.03	34.76 ± 22.94	0.424
Alanine transaminase	34.76 ± 22.94	62.17 ± 74.60	0.028
Gamma-Glutamyltransferase	44.05 ± 34.49	84.60 ± 97.36	0.082
Lactate Dehydrogenase	293.5 ± 116.1	517.0 ± 447.5	0.0001
Albumin	3.891 ± 0.403	3.214 ± 0.480	<0.0001
Days of hospital stay	10.14 ± 6.204	19.14 ± 11.48	<0.0001
Survival	35 (92.10%)	18 (47.36%)	<0.0001

## Data Availability

All study-relevant data are included in the current manuscript.
